# Predictive value of prognostic nutritional index for the long-term prognosis of elderly patients with fracture: a systematic review and meta-analysis

**DOI:** 10.3389/fnut.2025.1631128

**Published:** 2025-08-11

**Authors:** Bing Bai, Xilin Liu, Hong Li

**Affiliations:** ^1^Medical Service Dimension, China-Japan Union Hospital of Jilin University, Changchun, China; ^2^Department of Hand and Foot Surgery, China-Japan Union Hospital of Jilin University, Changchun, China; ^3^Department of Nursing, China-Japan Union Hospital of Jilin University, Changchun, China

**Keywords:** prognostic nutritional index, elderly, fracture, mortality, meta-analysis

## Abstract

**Objectives:**

To present the most recent and comprehensive systematic review and meta-analysis assessing the predictive value of prognostic nutritional index (PNI) for the long-term outcomes of elderly patients with fractures.

**Methods:**

We systematically searched PubMed, Cochrane, Embase and Web of Science up to July, 2025 for studies assessing the predictive value of PNI for the long-term prognosis of elderly patients with fractures. The primary outcome measured was mortality. Odds ratios (ORs) and 95% confidence intervals (CIs) were used for data pooling. Additionally, sensitivity and subgroup analyzes were performed to assess the stability of the results and identify potential sources of heterogeneity. All analyzes were performed using Review Manger 5.4 and STATA 15.1.

**Results:**

Eight studies encompassing 11,576 elderly individuals were included for meta-analysis. The meta-analysis demonstrated that mortality was notably lower in individuals with high PNI compared to those with low PNI (OR: 0.75; 95% CI: 0.66, 0.85; *p* < 0.0001). Subgroup analysis of mortality based on study design and PNI cut-off showed that the predictive value of PNI for mortality remained significant in prospective studies and those with a cut-off value ≥40. However, this association was not observed in retrospective studies or those with a cut-off below 40.

**Conclusion:**

PNI can effectively predict the long-term mortality in elderly individuals with fractures. Considering the limitations of this article, future large-scale, multicenter prospective cohort studies are still required to evaluate the prognostic value of PNI in senior patients with fractures and its influencing factors.

**Systematic review registration:**

PROSPERO, identifier CRD420251047385.

## 1 Introduction

Elderly people are more prone to falls and fractures due to decreased coordination, comorbidities, and increased bone fragility (1). Because their recovery ability is not as good as that of young people, fractures can seriously affect the physical and mental functions of elderly patients (2). The health-related quality of life and health status of most elderly patients do not fully return to pre-fracture levels (3). Among them, hip fracture is one of the most common types of fractures in the elderly population, with high disability and mortality rates (4, 5). It is estimated that by 2050, China will fully enter an aging society, and the number of hip fracture cases in China is expected to increase sixfold (6). Approximately 40 to 60% of elderly individuals experience hip fractures, and the mortality rate in the first 30 days after fracture is as high as 8–10%, and one-year mortality rate approaching 20–28% (2, 6). Therefore, exploring the factors influencing poor prognosis in elderly individuals with fractures following surgery is crucial to achieving early prediction and mitigating risk factors to improve patient outcomes.

Multiple studies have found that poor nutritional status plays a significant role in poor prognosis after surgery among senior fracture patients. It can also lead to the occurrence of refeeding syndrome and increase the mortality risk in patients (7–9). Calculated using peripheral blood lymphocyte counts and serum albumin concentrations, the prognostic nutritional index (PNI) is a key indicator of the body’s nutritional and immune status (10, 11). Initially, it was designed to assess the nutritional and immune status of patients undergoing gastrointestinal surgery. Recently, there has been a growing body of research highlighting the prognostic value of PNI in elderly patients with fractures (12–14). Some studies have suggested that PNI levels serve as a reliable indicator for evaluating malnutrition, and low levels of PNI are associated with higher mortality and worse postoperative mobility in elderly patients with fractures, and have important predictive value for long-term poor prognosis (15–17). In addition, PNI was found to be an independent predictor of postoperative intensive care unit requirement in patients with hip fractures (18). However, due to differences in sample size, follow-up time, treatment methods, and fracture sites, there is currently no definite conclusion on whether PNI can effectively predict the long-term prognosis of elderly individuals with fractures.

Although Liu et al. (19) conducted a meta-analysis to assess the role of PNI for postoperative mortality among individuals with hip fracture, they only included 3 original studies and revealed no significant link between PNI levels and mortality in hip fracture patients. After the publication of Liu et al.’s study, numerous new clinical studies have been conducted to assess the long-term prognostic value of PNI in elderly fracture patients, and the conclusions do not support previous studies (15–18, 20–22). Therefore, this study focuses on PNI as an easily measurable peripheral circulation marker. Through systematic literature retrieval and meta-analysis, it expands upon previous meta-analyzes by incorporating the latest research data to further evaluate PNI’s ability to predict the long-term prognosis of elderly individuals with fractures. This study aims to provide updated evidence-based insights for constructing a prognostic prediction model for elderly fracture patients.

## 2 Methods

### 2.1 Literature search

Following the PRISMA 2020 statement (Preferred Reporting Items for Systematic Reviews and Meta-Analysis), this meta-analysis was performed and prospectively registered in the PROSPERO database (CRD420251047385). A systematic literature search was conducted across PubMed, Embase, Web of Science, and Cochrane up to July 2025 to collect studies focusing on the prognostic role of PNI in the long-term outcomes of elderly fracture patients. The literature was searched via the following keywords: “prognostic nutritional index,” “PNI,” and “fracture,” etc. The search strategies used in PubMed are outlined below: ((“Fractures, Bone”[Mesh]) OR (((((((((Bone Fracture) OR (Bone Fractures)) OR (Broken Bones)) OR (Broken Bone)) OR (Spiral Fractures)) OR (Spiral Fracture)) OR (Torsion Fractures)) OR (Torsion Fracture)) OR (Fracture))) AND ((prognostic nutritional index[Title/Abstract]) OR (PNI[Title/Abstract])). Furthermore, the reference lists of the selected studies were manually examined. Two authors identified and assessed qualifying articles separately, and any disagreements were addressed by discussion (show in Supplementary Table S1).

### 2.2 Inclusion and exclusion criteria

The following conditions determined eligibility for articles: (1) the study design was a randomized controlled trial, cohort, or case–control; (2) the studies were conducted in elderly individuals with fractures; (3) the studies assessed the role of PNI in long-term prognosis (at least 3 months after the fracture) of elderly fracture patients; (4) at least one long-term outcome was evaluated, including mortality, refracture, cardiovascular event, etc.; (5) sufficient data to analyze odds ratio (OR). Exclusions included review articles, non-original studies (such as letters, comments, abstracts, corrections, and replies), study protocols, unpublished articles, and studies with inadequate data.

### 2.3 Data abstraction

Data abstraction was performed independently by two authors, with any differences resolved through discussion with a third author. The following details were extracted from the qualified studies: first author name, published year, study country, study design, types of fractures, sample size, age, gender, BMI, PNI cut-off, and outcomes. In cases of insufficient research data, corresponding authors were contacted to obtain the complete data, if available.

### 2.4 Quality evaluation

The Newcastle-Ottawa Scale (NOS) was used to evaluate the quality of the included cohort studies (23), and studies scoring 7–9 points were deemed high quality (24). Two authors independently reviewed the quality of each study, with any differences addressed through discussion.

### 2.5 Statistical analysis

The meta-analysis was performed using Review Manager 5.4.1 software. OR and 95% confidence intervals (CIs) were applied for the data synthesis. To evaluate the heterogeneity of each outcome, the chi-squared (χ^2^) test (Cochran’s Q) and I2 index were employed (25). χ^2^ *p* value below 0.1 or *I*^2^ value exceeding 50% was considered indicative of high heterogeneity. The random-effects model was then utilized to estimate the overall OR for all outcomes. Additionally, subgroup analyzes were conducted to assess possible confounding factors, provided sufficient data were available. Furthermore, a sensitivity analysis was conducted to assess the impact of each included study on the overall odds ratio (OR) for each outcome. Publication bias was assessed by generating funnel plots using Review Manager 5.4.1 and performing Egger’s regression tests (26) with Stata 15.1 (Stata Corp, College Station, Texas, United States). A *p*-value < 0.05 was considered indicative of statistically significant publication bias.

## 3 Results

### 3.1 Literature retrieval, study characteristics, and baseline

**Figure 1:**
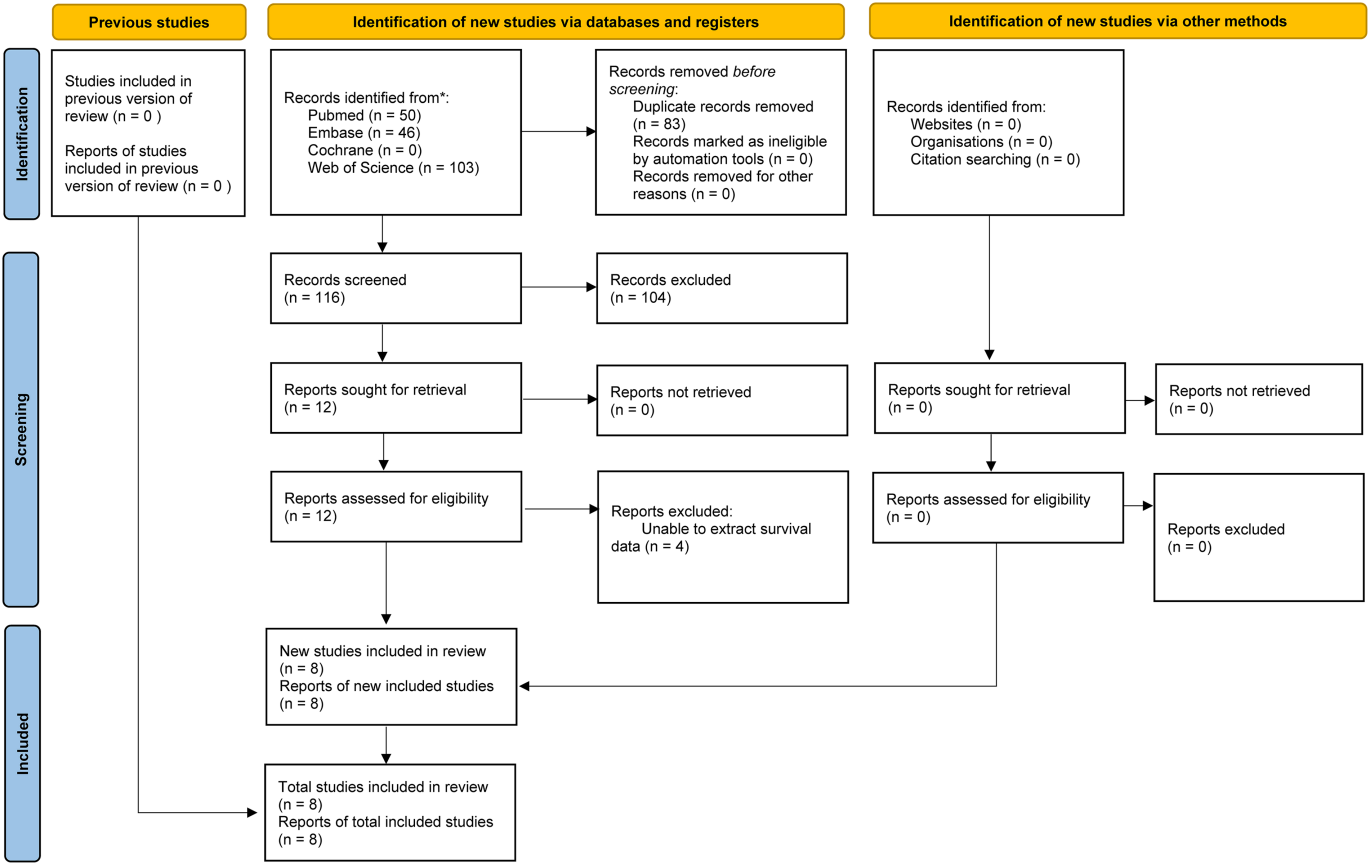
Flowchart of the systematic search and selection process.

The literature retrieval and selection process is illustrated in the flowchart presented in Figure 1. A total of 199 relevant studies were identified through a systematic literature search across PubMed (n = 50), Embase (n = 46), Web of Science (n = 103), and Cochrane (n = 0). After removing duplicates, 116 titles and abstracts were reviewed. In the end, 8 cohort studies (with 11 comparative groups) comprising 11,576 patients were incorporated into the meta-analysis (15–18, 20–22, 27). The details and quality assessment of each included cohort study are provided in Table 1.

**Table 1:** Characteristics and quality assessment of included studies.

Author	Region	Study design	Types of fractures	No. of patients	Gender		Mean/median age	Mean/median BMI	PNI cut-off	Nos score
					Male	Female				
Arslan 2024 (18)	Turkey	Retrospective	Hip fracture	222	75	147	80.6	25.9	32.5	7
Arslan 2025 (25)	Turkey	Retrospective	Orthopedic trauma	371	142	229	78.5	25.5	26	7
Chen 2024a (13)	China	Prospective	Hip fracture	942	261	681	79.6	23	43.55	7
Chen 2024b (16)	China	Prospective	Hip fracture	942	261	681	79.6	23	46.55	7
Chen 2024c (37)	China	Prospective	Hip fracture	942	261	681	79.6	23	49.2	7
Feng 2021 (22)	China	Prospective	Hip fracture	221	58	163	78	NA	NA	8
Feng 2022 (21)	China	Retrospective	Hip fracture	195	51	144	78	23	38	7
Tunçez 2024 (15)	Turkey	Prospective	Femoral neck fracture	124	47	77	80.4	26.8	38.4	8
Wang 2023 (17)	China	Retrospective	Hip fracture	3,351	1,118	2,233	80	23	43.23	7
Wang 2023 (17)	China	Retrospective	Hip fracture	3,351	1,118	2,233	80	23	47.45	7
Yılmaz 2024 (20)	Turkey	Retrospective	Hip fracture	915	345	570	81.09	NA	34.475	7

### 3.2 Mortality

**Figure 2:**
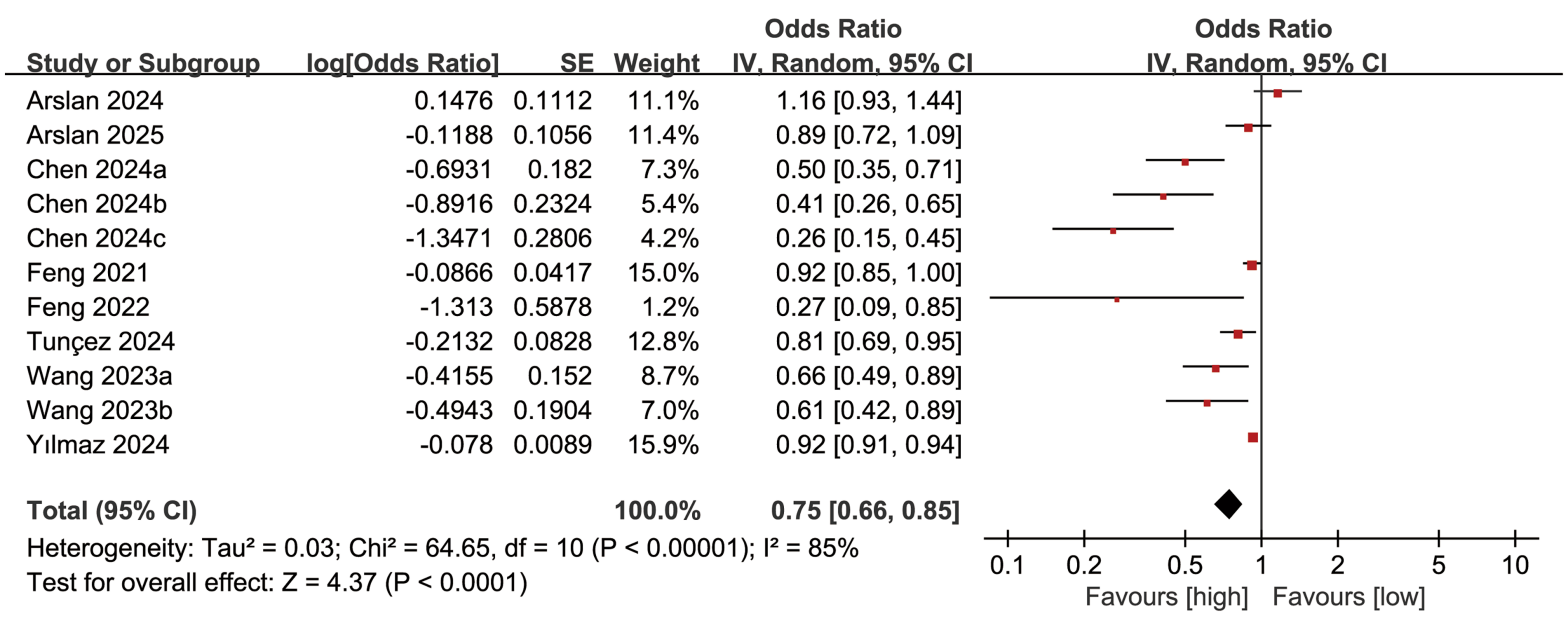
Forest plots of mortality.

The mortality outcomes were derived from 8 cohort studies, and the meta-analysis indicated a significantly reduced mortality rate in the high PNI group compared to the low PNI group (OR: 0.75; 95% CI: 0.66, 0.85; *P* <0.0001). Significant heterogeneity was identified (*I*^2^ = 85%, *P* <0.00001) (Figure 2).

### 3.3 Publication bias and sensitivity analysis

**Figure 3:**
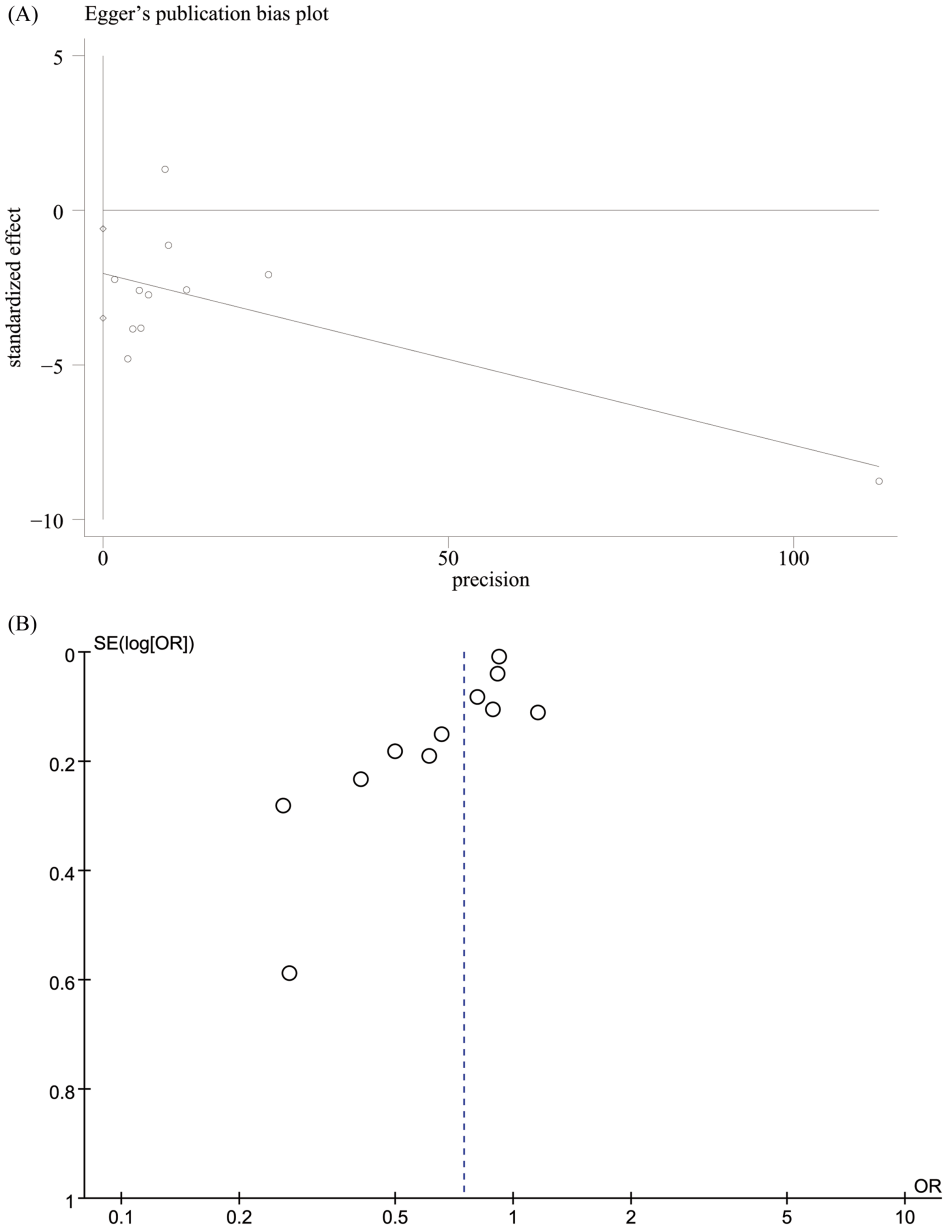
Egger’s test plots **(A)** and funnel plots **(B)** of mortality.

**Figure 4:**
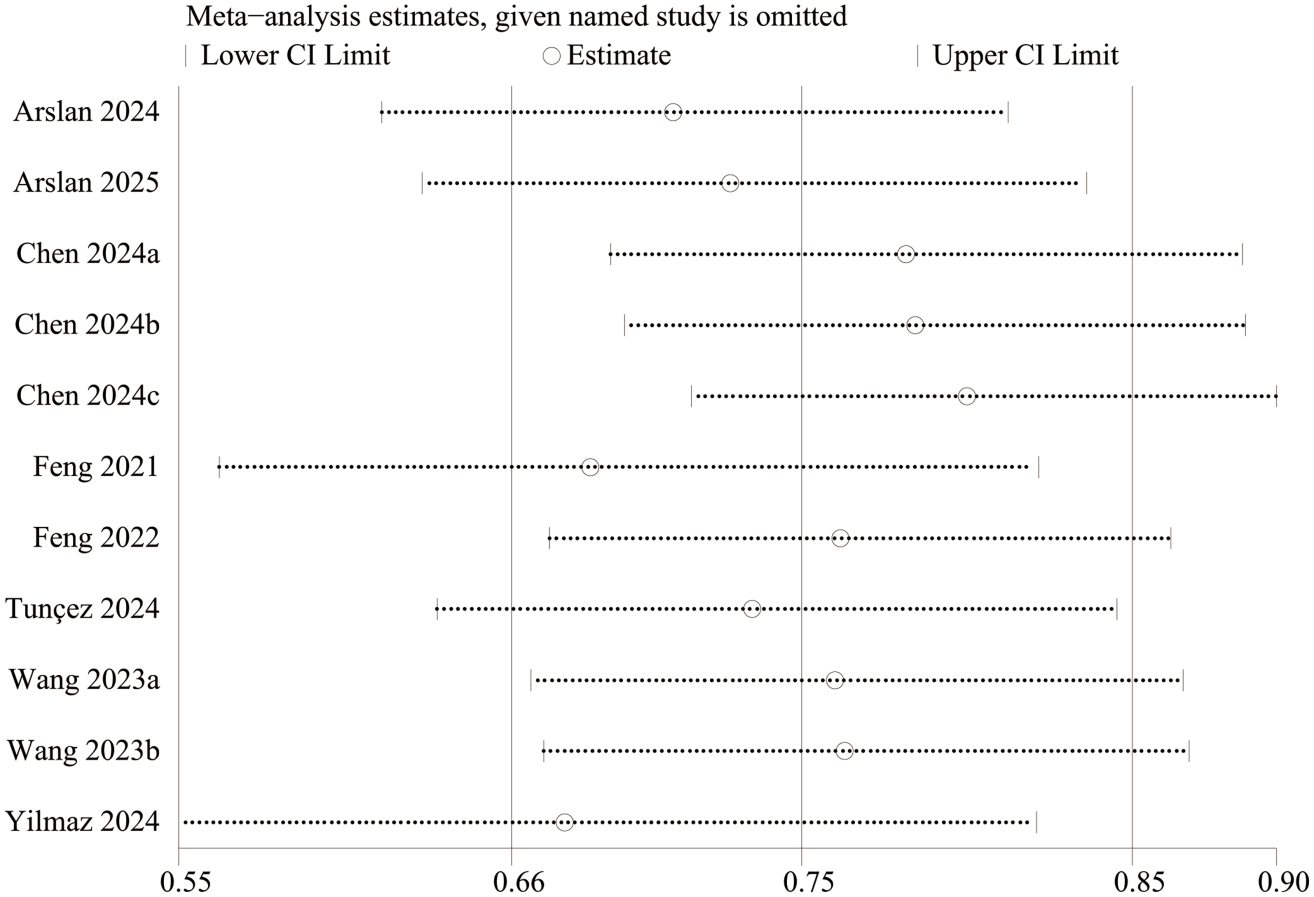
Sensitivity analysis of mortality.

Funnel plots and Egger’s regression tests were used to identify potential publication bias for mortality. Egger’s test (*p* = 0.012, Figure 3A) and funnel plots (Figure 3B) detected a significant publication bias for mortality. We also performed a sensitivity analysis on the mortality outcomes to assess the influence of each cohort study on the overall OR by sequentially removing studies. The results demonstrated that the total OR remained consistent after the removal of each study for mortality (Figure 4).

### 3.4 Subgroup analysis

**Figure 5:**
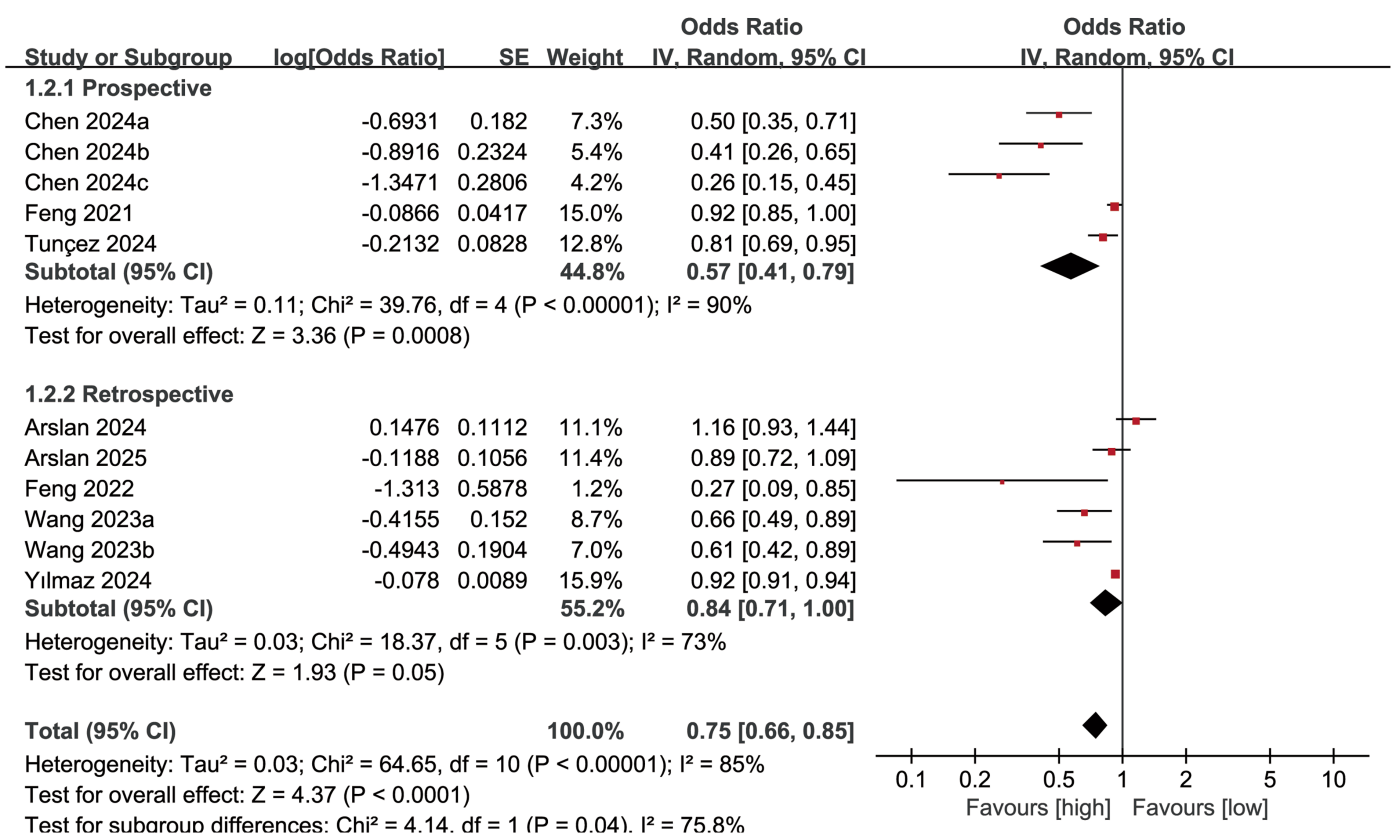
Subgroup analysis of mortality based on study design.

**Figure 6:**
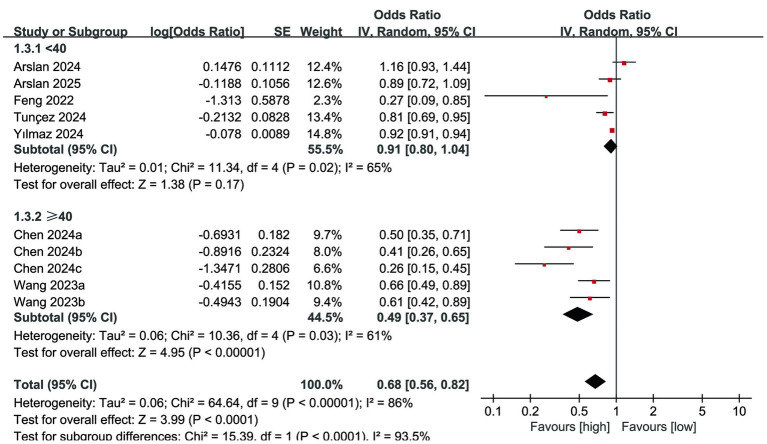
Subgroup analysis of mortality based on PNI cut-off.

Subgroup analysis of mortality according to study design showed that the predictive value of PNI for mortality continued to be significant in prospective studies (OR: 0.57; 95% CI: 0.41, 0.79; *p* = 0.0008) but disappeared in retrospective studies (OR: 0.84; 95% CI: 0.71, 1.00; *p* = 0.05) (Figure 5). At the same time, a decrease in heterogeneity was observed in retrospective studies. Subgroup analysis of mortality according to PNI cut-off values showed that the predictive value of PNI for mortality continued to show significance in studies with a cut-off value ≥ 40 (OR: 0.49; 95% CI: 0.37, 0.65; *P* <0.00001), but disappeared in studies with a cut-off value < 40 (OR: 0.91; 95% CI: 0.80, 1.04; *p* = 0.17) (Figure 6). At the same time, a decrease in heterogeneity was observed in both subgroups.

## 4 Discussion

Studies have pointed out that elderly patients with fractures have a higher risk of malnutrition during the perioperative period, which will increase the mortality rate of patients. The main reasons are that the elderly have decreased body function, decreased appetite, reduced nutritional intake, and often have chronic diseases, physiological and psychological problems (28). PNI is a commonly used indicator for clinical evaluation of preoperative nutritional status, surgical risk and prognosis of surgery, and has certain value in predicting the prognosis of elderly individuals with fractures. Katsuyama et al. (29) included 1,026 hip fracture patients aged 65 years or older who had undergone surgery. The results showed that PNI was an important predictor of postoperative mortality in individuals with hip fractures, but this conclusion was not supported by all studies (18, 21). Therefore, this article further evaluated the role of PNI in predicting the long-term prognosis of elderly fracture patients, providing up-to-date evidence to inform the creation of a prognosis prediction model for this population.

This study found that PNI was significantly associated with long-term mortality after surgery in senior fracture patients, and the lower the PNI, the higher the mortality rate of the patient. Sensitivity analysis further verified that this result was not significantly affected by a single study, and the evidence was stable and reliable. However, it is worth noting that a significant publication bias was detected in mortality, which could potentially influence the reliability of the findings to some extent. We speculate that regional selection bias may be one of the main causes of publication bias, because the literature included in this study are all studies in Asia, mainly China and Turkey, which also needs to be further resolved by international multicenter studies. In addition, significant heterogeneity is also a problem that cannot be ignored in this study. Based on this, we conducted a subgroup analysis based on the study design and PNI cut-off value, and the results suggested that the significant heterogeneity of mortality can be partially explained by the study design and PNI cut-off value.

Our conclusions do not support the meta-analysis results of Liu et al. (19), which suggested that there is no significant link between PNI and mortality in individuals with hip fractures. It is worth noting that the meta-analysis of Liu et al. only included 3 original studies, which resulted in unavoidable small sample size effects and selection bias. In addition, the study of Liu et al. did not conduct rigorous sensitivity analysis and subgroup analysis, and it is impossible to determine whether its results would be significantly affected by a single study or a certain factor. Therefore, the existing evidence supports that PNI can significantly predict the mortality of elderly patients with fractures, but additional research is required to validate this.

On the other hand, our mortality subgroup analysis according to the study design showed that the prognostic value of PNI for mortality was still relevant in prospective studies, but disappeared in retrospective studies. This differential result does not change the overall quality of this study, because it is well known that the quality of evidence in prospective studies is better than that in retrospective studies. In addition, the subgroup analysis of mortality according to the PNI cut-off value showed that the predictive value of PNI for mortality was still significant in studies with a cut-off value ≥ 40, but disappeared in studies with a cut-off value < 40. However, there is no definite conclusion on the optimal cut-off value of PNI (14, 30). Different studies have different optimal cut-off value confirmation methods (although most of them are through ROC curves), and different fracture populations also have different optimal PNI cut-off values (12, 31, 32). The findings of this study are more inclined to determine the cut off value of PNI above 40 in order to maximize its predictive value, but this result should be validated through additional studies with larger sample sizes.

Elderly patients with fractures are often accompanied by malnutrition, which leads to decreased serum albumin levels and decreased lymphocyte counts (11, 33, 34). Serum albumin plays a crucial role in maintaining plasma osmotic pressure and transporting substances (35–37). Its decreased level can affect the body’s metabolism and immune function (37). As a crucial component of the immune system, a decrease in the number of lymphocytes can lead to decreased immune function and increase the risk of complications such as infection (31, 38). Therefore, PNI can indirectly reflect the patient’s nutritional and immune status by evaluating serum albumin and lymphocyte counts, thereby predicting their postoperative recovery and prognosis (39, 40). In addition, in elderly patients with fractures, disorders of the endocrine system may lead to abnormal metabolism and decreased immune function (41). For example, insulin resistance and decreased growth hormone secretion may lead to decreased protein synthesis and increased decomposition, further aggravating malnutrition (42, 43). As an indicator for evaluating nutritional and immune status, PNI may reflect the regulatory function of the endocrine system to a certain extent. Furthermore, the regulation of the nervous system may be affected in elderly patients with fractures, resulting in a decrease in the body’s ability to regulate nutritional and immune status (44). For example, disorders of the autonomic nervous system may lead to gastrointestinal dysfunction and malnutrition (45, 46). As an indicator for evaluating nutritional and immune status, PNI may reflect the regulatory function of the nervous system to a certain extent.

Nevertheless, it is important to recognize some limitations of this analysis. Owing to the nature of clinical research, a significant portion of the studies included are retrospective cohort studies. As is well-known, confounding factors and risk of bias are the biggest disadvantages of retrospective studies. In addition, the literature analyzed in this meta-analysis predominantly comes from Asia, especially China and Turkey, and lacks data from Europe and the United States. Therefore, the generalizability of these results to other countries is not clear. Lastly, the results of PNI predicting mortality in elderly patients with fractures have obvious heterogeneity and publication bias, which have not been fully explained. Therefore, it is important to be cautious when interpreting the relationship between PNI and mortality in elderly patients with fractures. Despite these limitations, this analysis represents the most recent largest study reporting the prognostic value of PNI in elderly fracture patients. The results underscore the significance of monitoring PNI levels during the clinical management of these patients. Moreover, they highlight the potential of developing a more robust fracture prognosis prediction model incorporating inflammatory markers like PNI, ultimately aiming to enhance patient outcomes and their quality of life.

## 5 Conclusion

As a clinically accessible, inexpensive, and noninvasive nutritional marker, PNI can effectively predict the long-term mortality of senior fracture patients, and help to identify high-risk individuals with poor prognosis at an early stage and take targeted preventive and treatment measures. Subgroup analysis found that using a PNI cutoff value of 40 or higher provides better predictive value. Considering the limitations of this article, such as significant heterogeneity, publication bias, and regional selection bias, there is still a need for more extensive, multicenter prospective cohort studies to evaluate the prognostic value of PNI in elderly fracture patients and its influencing factors.

## Data Availability

The original contributions presented in the study are included in the article/Supplementary material, further inquiries can be directed to the corresponding author.
