# Supplementary material for: Predictive value of prognostic nutritional index for the long-term prognosis of elderly patients with fracture: a systematic review and meta-analysis
Source: Front Nutr. 2025 Aug 11;12:1631128. doi: 10.3389/fnut.2025.1631128 (PMC12375473; doi:10.3389/fnut.2025.1631128)
Supplement: Supplementary file 1 [file Table_1.docx]

TableS1 Literature Search Strategy

Pubmed-45

(("Fractures, Bone"[Mesh]) OR (((((((((Bone Fracture) OR (Bone Fractures)) OR (Broken Bones)) OR (Broken Bone)) OR (Spiral Fractures)) OR (Spiral Fracture)) OR (Torsion Fractures)) OR (Torsion Fracture)) OR (Fracture))) AND ((prognostic nutritional index[Title/Abstract]) OR (PNI[Title/Abstract]))

Embase-34

((Fractures, Bone or (Bone Fracture or Bone Fractures or Broken Bones or Broken Bone or Spiral Fractures or Spiral Fracture or Torsion Fractures or Torsion Fracture or Fracture)) and (prognostic nutritional index or PNI)).ab.

Cochrane-0

((Fractures, Bone or (Bone Fracture or Bone Fractures or Broken Bones or Broken Bone or Spiral Fractures or Spiral Fracture or Torsion Fractures or Torsion Fracture or Fracture)) and (prognostic nutritional index or PNI)).ab.

WOS-83

((Fractures, Bone) OR (((((((((Bone Fracture) OR (Bone Fractures)) OR (Broken Bones)) OR (Broken Bone)) OR (Spiral Fractures)) OR (Spiral Fracture)) OR (Torsion Fractures)) OR (Torsion Fracture)) OR (Fracture))) AND ((prognostic nutritional index) OR (PNI)) (Topic)
